# Adolescents' Experiences of Social Media Content Types and Wellbeing: A Photo‐Elicitation Interview Study

**DOI:** 10.1002/jad.70231

**Published:** 2026-07-14

**Authors:** Lizzy Winstone, James Parsonage, Cassey Muir, Amie Randhawa, Lauren Cross, Georgie MacArthur, Evangelina Asiedu‐Addo, Emma Garavini

**Affiliations:** ^1^ Population Health Sciences, Canynge Hall University of Bristol Bristol UK; ^2^ Population Health Sciences Institute Newcastle University Newcastle UK; ^3^ School of Sport, Exercise and Rehabilitation Sciences University of Birmingham Birmingham UK; ^4^ MRC Epidemiology Unit University of Cambridge Cambridge UK; ^5^ The McPin Foundation Young People's Network The McPin Foundation London UK

**Keywords:** adolescents, algorithmic recommendation, mental health, photo‐elicitation, qualitative research, social media content, wellbeing

## Abstract

**Background:**

Adolescents' experiences of social media are increasingly shaped by algorithmic recommendation systems that structure repeated exposure to diverse content types. However, research has focused predominantly on screen time, with limited attention to the diverse types of content encountered within curated feeds or how these experiences intersect with developmental processes such as identity formation, social evaluation, and emotional regulation.

**Methods:**

Semi‐structured photo‐elicitation interviews were conducted in 2025 with 27 adolescents aged 14–19 years in the United Kingdom (mean age = 17.15 years, SD = 1.49; 70% self‐described female). Participants shared screenshots from their TikTok For You or Instagram Explore pages to support discussion of algorithmically recommended content. Data were analysed using reflexive thematic analysis.

**Results:**

Seven content types were identified: entertainment, consumerism, identity‐related, mental health, news and activism, body image, and AI‐generated content. All were experienced as double‐edged, with perceived benefits and risks shaped by repeated algorithmic exposure, developmental stage, emotional state, and individual context. Participants frequently reflected retrospectively on earlier social media experiences, identifying younger adolescence as a period of particular vulnerability. Perceived impacts were closely connected to developmental processes including identity formation, social evaluation, and emotional regulation, with younger adolescents perceived as less equipped to critically evaluate content or manage algorithmic exposure.

**Conclusions:**

Adolescents' wellbeing in relation to social media appears shaped by the types of content repeatedly encountered and the developmental context in which exposure occurs. Research, education, and platform design should address the quality, recurrence, and emotional consequences of algorithmically curated content exposure across adolescence.

## 1 Introduction

Adolescence is a period of significant developmental change, characterised by identity development, greater sensitivity to peers and social evaluation, increasing independence, and continued maturation of emotional regulation and higher‐order thinking skills (Blakemore 2019). During this period, adolescents become more responsive to social approval and rejection, and more influenced by peer norms, while capacities such as planning, inhibition, and emotion regulation are still developing. These changes shape how social information is interpreted and responded to, increasing the salience of online environments that provide continuous social feedback through likes and comments (McGorry et al. 2024). As a result, digital environments are increasingly recognised as integral to adolescent development, shaping how young people experience identity, peer relationships, and emotional processes in everyday life (Orben and Blakemore 2023; Orben et al. 2024).

A substantial body of developmental research has examined how peer relationships and social influence shape adolescent behaviour and wellbeing (Nesi et al. 2018a, 2018b, 2020). Research suggests that social media transforms these peer processes by increasing the frequency, visibility, and intensity of social interactions, and by amplifying exposure to peer norms and feedback (Nesi et al. 2020). However, this research has largely focused on interpersonal interactions and peer relationships, with less attention to how algorithmically curated systems structure the content adolescents are repeatedly exposed to within these digital environments, potentially also transforming processes of identity and emotional development. Alongside this, debates about social media and wellbeing have often focused on how much time adolescents spend online, rather than what they are shown. Adolescent experiences of social media are increasingly shaped not only by their own choices, but by recommendation systems that prioritise and repeat particular forms of content (Perez Vallejos et al. 2021). Platforms such as TikTok and Instagram organise ongoing personalised exposure to highly diverse content, which may evoke different emotional, cognitive, and behavioural responses. Despite this, much research continues to rely on broad measures of screen time, failing to differentiate between different online experiences or types of content exposure (Odgers and Jensen 2020).

Emerging evidence increasingly suggests that understanding social media effects requires attention to developmental stage and to the specific types of experiences adolescents encounter online (Orben and Blakemore 2023). However, little is known about how developmental processes such as social evaluation, identity formation, and emotional regulation unfold within algorithmically curated environments, where exposure to particular types of content is repeated, personalised, and often difficult to control. An affordances perspective proposes that the features of social media platforms shape adolescents' opportunities for interaction, self‐expression, identity exploration, and social feedback, and that these affordances should be understood alongside adolescent developmental needs and capacities (Moreno and Uhls 2019). Algorithmically driven content exposure may therefore represent a novel developmental context in which peer influence, social comparison, and identity‐related processes are organised and experienced in new ways (Nesi et al. 2018a). Recent ecological momentary assessment research demonstrates that adolescents experience a range of positive and negative social media experiences in everyday life, including entertainment, social connection, appearance‐based comparison, conflict, and exposure to upsetting content, with emotional wellbeing varying according to the nature of these experiences rather than overall social media use (Sequeira et al. 2025; Hamilton et al. 2024; Boyd et al. 2024). However, these studies typically characterise experiences at a broad level. Further insight is needed into the specific forms of algorithmically recommended content that produce them, how repeated exposure shapes their emotional significance, anhow adolescents themselves make sense of these experiences within their everyday feeds.

The Differential Susceptibility to Media Effects Model (DSMM, (Valkenburg and Peter 2013)) further emphasises that media effects depend on the interaction between individual susceptibilities, social and developmental contexts, and specific media features (Piotrowski and Valkenburg 2015). Understanding experiences of different types of social media content is therefore essential, as content characteristics shape emotional engagement and potential impacts on wellbeing. Some prior research has examined specific forms of content, such as self‐harm or body image, and findings suggest that impacts are context‐dependent and can be both beneficial and harmful (Sharma and Vidal 2023; Susi et al. 2023). However, little research has examined how adolescents experience multiple types of content within algorithmically curated feeds, in ways that may support or undermine wellbeing and key developmental tasks.

This study therefore examines how adolescents experience different types of algorithmically recommended content, and how these experiences relate to developmental processes including identity formation, social evaluation, and emotional regulation. Drawing on qualitative photo‐elicitation interviews, we explore the range of content adolescents encounter, the benefits and risks they associate with different content types, and the factors that shape when content is experienced as helpful, neutral, or harmful. We also examine how perceived impacts vary across developmental stages and changing capacities for critical evaluation and emotional regulation. This paper forms part of a wider qualitative project. A separate companion paper develops a grounded theory model of the socioecological influences of digital mental health within algorithmic systems (Winstone et al. 2026). In contrast, the present analysis focuses specifically on adolescents' lived experiences of content types and their perceived implications for wellbeing during everyday social media use.

## 2 Methods

The study is reported in accordance with the ‘Consolidated criteria for reporting qualitative studies’ (COREQ) checklist (Tong et al. 2007). Data collection and analysis took place in 2025.

### 2.1 Participants and Recruitment

Participants were recruited via the McPin Foundation Young People's Network, emails to PSHE leads or headteachers in UK secondary schools, and youth mental health and youth groups. Study materials and design were developed with input from six members of the #ForYou Young People's Advisory Group (McPin Foundation).

Twenty‐seven adolescents aged 14–19 years participated (mean age = 17.15 years, SD = 1.49). Participants provided age and self‐described gender identity. Nineteen (70%) identified as female, seven as male, and one as gender diverse. Recruitment through mental health networks aimed to include adolescents with experience of mental health difficulties; however, mental health experience was not formally captured as a participant characteristic. References to mental health in the analysis are based on experiences shared during interviews. Although demographic characteristics such as ethnicity, sexual orientation, transgender identity, and neurodiversity were not systematically collected, several participants spontaneously referenced these aspects of identity during interviews. These accounts included references to LGBTQ+ identities, transgender experiences, minority ethnic backgrounds, and neurodiversity. These categories were not mutually exclusive and are noted here to provide context of the diversity of experiences represented, rather than as formal sample characteristics.

### 2.2 Procedure and Data Collection

Participants attended a 15‐min online briefing prior to interview. The day before the interview, participants shared 2–10 screenshots from their TikTok *For You* page or Instagram *Explore* page, showing algorithmically recommended content that was typical or meaningful to them.

Semi‐structured interviews were guided by flexible use of a topic guide (Supporting Information). This was co‐developed with the youth advisory group, and covered social media use, emotionally salient content, and retrospective reflection of changes in online content experiences over time. Screenshots were discussed in turn, including participants' perceptions of why content was recommended and its emotional impact. As analysis progressed, the guide was refined to include interpretations of healthy and harmful social media use and greater attention to school, peer, and family influences. Interviews lasted approximately 1 hour, were audio‐recorded, and transcribed verbatim.

Ethical approval was granted by the University of Bristol Faculty of Health Sciences Research Ethics Committee (ref: 22479). All participants gave informed consent prior to participation, with parental or guardian consent and participant assent obtained for those under 16 years. Given the sensitivity of discussing personal feeds and potentially distressing content, participants were reminded they could pause or stop interviews at any time. Care was taken to anonymise screenshots by obscuring account names and faces. Following the interview, participants were signposted to sources of support relating to online safety and mental health. Participants received a £25 shopping voucher in recognition of their time.

### 2.3 Researchers and Reflexivity

Interviews were conducted online between March and September 2025 by LW (female, PhD, Senior Research Associate), an experienced qualitative researcher with expertise in digital mental health, and JP (male, MSc, Pre‐doctoral Fellow in public health research), who was trained but new to qualitative interviewing. Participants did not have knowledge of the research aims. Reflective notes were written after each interview. We approached analysis with a critical realist position, treating participants' accounts as real and meaningful lived experiences whilst acknowledging them as socially and contextually shaped and interpreted (Braun and Clarke 2019).

### 2.4 Data Analysis

Reflexive thematic analysis was conducted to construct and develop themes through iterative interpretation of patterns across participants' accounts (Braun and Clarke 2019). Data familiarisation and initial inductive coding was undertaken independently by LW, JP, LC, and six youth advisers to encourage multiple interpretive perspectives. Coding was subsequently discussed in a series of analytic meetings, during which similarities, differences and alternative interpretations were explored to deepen conceptual understanding. Rather than calculating inter‐rater reliability, discussions were used to deepen interpretation, consistent with Braun and Clarke's approach to reflexive thematic analysis (Braun and Clarke 2019, 2020). Codes were first developed at a semantic level before being iteratively grouped into initial themes based on team discussions of shared meaning and patterns across participants. Via team discussions, themes were refined through repeated comparison across transcripts, with attention to variation, contradictions, and developmental differences in participant experiences. Data were managed using NVivo 14. Analysis involved active interpretation of how adolescents made sense of content in relation to their emotional responses and developmental context, developing an explanatory account of shared patterns across participants. Retrospective accounts were analysed alongside current reflections as a source of insight into developmental change.

## 3 Results

Participants described encountering a wide range of social media content types within algorithmically curated feeds. Across all themes, content was experienced as double‐edged, with perceived benefits and risks shaped by repeated exposure and individual context. A recurring feature of participants' accounts was reflection on their own earlier social media experiences, often identifying younger adolescence as a period of particular vulnerability in relation to concerns that are especially salient during this developmental period, including appearance, peer norms, and emotional coping. These retrospective perspectives are woven throughout the themes below and offer insight into how experiences of algorithmically curated content may shift as regulatory and evaluative capacities develop across adolescence. Table 1 summarises the major content types, their perceived affordances, risks, and contextual influences (see Supplementary Table 1 for a summary of themes, descriptive codes and illustrative data extracts).

**Table 1:** Adolescents' perceived benefits, risks, and contextual influences across social media content types.

Content type	Positive affordances	Risks	Conditions influencing impact
Entertainment	Relief, enjoyment, distraction, mood regulation, connection through shared humour/interests	Time displacement, compulsive scrolling, guilt, reduced productivity	Frequency of exposure, boredom, stress, habitual use
Consumerism	Product discovery, inspiration, convenience, identity expression	Pressure to consume, financial comparison, normalisation of overconsumption, ethical and environmental concerns, blurred advertising boundaries	Repeated exposure, commercial embedding within lifestyle content, financial circumstances
Identity‐related content	Representation, belonging, validation, community, identity exploration	Exposure to hate, stereotyping, conflict, discrimination, hostile comments	Offline support, comment culture, platform moderation, engagement with contentious content
Mental health content	Validation, awareness, coping ideas, recovery support, reduced isolation	Triggering content, rumination, reinforcement of distress, overwhelming	Mood, vulnerability, recovery stage, repeated exposure, literacy
News and activism	Awareness, civic engagement, empathy, moral responsibility, connection to wider world	Distress, helplessness, guilt, mistrust, overwhelming	Volume of exposure, graphic content, personal relevance, developmental stage
Body image	Inspiration, motivation, body positivity, self‐expression	Social comparison, internalisation of ideals, lower self‐esteem, objectification	Existing self‐esteem, age, repeated exposure across feeds, susceptibility to appearance norms
AI‐generated content	Curiosity, novelty, entertainment, creativity	Confusion, misinformation, mistrust, unease, ethical and environmental concerns	Ability to recognise AI, rapid scrolling, repeated exposure, perceived relevance

### 3.1 Entertainment as Relief and Time Displacement

Entertainment content included arts and culture, sports, cars, animals, lifestyle, and uplifting stories or memes. This formed a contrasting content type, functioning both as emotional relief and a mechanism of time displacement. Participants described humorous, cute, or light‐hearted content as offering short‐term mood regulation, distraction, and enjoyment, particularly during periods of stress or boredom.

I use it as a distraction, really, whenever I need to, or whenever I'm bored or whenever I'm not feeling okay, I just go on TikTok and scroll. I'm hoping to get some cute videos, so things that interest me generally.

Female, 19 years

So social media, like, when I was having a bereavement and mental health problems, it will just distract me from… what's going on in the world. So social media is like a mini platform to escape reality…

Male, 17 years

Algorithms were perceived to repeatedly amplify entertaining content due to its high engagement and sharing rates, creating feeds dominated by easily consumable material. This same process facilitated extended scrolling, with participants reflecting on how time spent engaging with entertainment content sometimes led to feelings of guilt, emptiness, or missed opportunities offline.

…the negatives to all that funny greatness is it's time consuming. Like you just spent two hours scrolling on TikTok but I could have done so much more stuff. I think the algorithm knows that I like cars so they put them type of videos to engage me and for me to pay attention to spend time on. I mean, the algorithm is just there to put good videos and make you hooked or like make you watch them. They're doing their job well… but it has a negative effect on you as a person.

Male, 15 years

Several participants reflected that extended scrolling had become more habitual over time, suggesting that algorithmically reinforced patterns of entertainment consumption may become increasingly entrenched across adolescence. Entertainment appeared to operate as a low‐demand sanctuary within social media environments, providing relief while also subtly and sometimes insidiously displacing engagement with other activities often seen by adolescents as more productive. Such tensions between immediate reward and longer‐term goals may reflect the ongoing development of self‐regulation during adolescence.

### 3.2 Consumerism as Convenience and Normalised Pressure

Consumerism appeared to be a pervasive content type through which algorithmically structured exposure embedded commercial influence into everyday social media use. This was sometimes perceived as benign or convenient, but some participants reflected on the ethical or environmental implications of overconsumption or exploitative product promotion.

I mean, it's a bit upsetting, isn't it? Like, if you see people buying thousands of pieces of clothes and just random plastic tat advertised to you, it just feels like not something I want to see, and I mean, it's terrible for the environment.

Female, 18 years

Participants described how algorithmic recommendation normalised consumption over time by repeatedly integrating advertising and product promotion into non‐commercial contexts, such as beauty routines, lifestyle content, hobbies, or entertainment. Some also perceived commercial content as deliberately using attractive people or visually engaging imagery to capture attention before transitioning into advertising, further blurring the boundaries between entertainment and marketing.

It didn't interest me at all, but you see a lot of stuff like that now, where it's just some girl or whatever. It's a way of attracting people to watching videos, and it might not be the same video. They might start with one to grab your attention, and then it could be like an advert for, like, a takeaway or something.

Male, 19 years

This blurring of boundaries made commercial messaging often feel subtle and routine rather than overt, contributing to perceptions of inevitability in encountering consumerist content while scrolling. Over time, repeated exposure created a background pressure around purchasing and self‐improvement, even when users did not actively seek out adverts.

If it's something about different products they were using for hair that I might want to try, then I would either save or like the post. And then I would try to order it.

Female, 18 years

Commodity‐related social comparison or affordability was occasionally interpreted as a point of tension, with some participants describing stress when recommended products felt financially out of reach.

Back in COVID, when I used to look at a lot of this [money‐related] content, it would make me think, “Oh, why do I not have it? How can I get it?”

Male, 17 years

Through algorithmically patterned exposure, consumption was positioned as a social norm or expectation, illustrating how content types can exert influence without requiring deliberate engagement.

### 3.3 Identity Content as Belonging and Conflict

Identity‐related content simultaneously offered affirmation and exposure to conflict and prejudice. Algorithms were perceived to cluster content around aspects of identity such as gender, sexuality, culture, neurodivergence, or interests. This was repeatedly described as fostering recognition, relatability, and a sense of belonging. For some participants, this content appeared to support identity exploration and connection during an important developmental period in which questions of self‐definition and peer acceptance are especially salient (Branje et al. 2021).

I follow a lot of queer accounts… I watch their videos about their relationship and their queer experiences and stuff. And so, yeah, I tend to have a lot of LGBTQ things on my feed.

Female, 16 years

However, several participants described how the same engagement‐driven processes that they perceived to amplify supportive content were also felt to intensify exposure to hostility, stereotypes, and discriminatory comments.

It [content showing the Bengali flag] makes me feel seen, but then the comments can be quite hateful… like a little war between countries… and people just hating on each other.

Female, 19 years

Participants also noted that content relating to women and gender roles could attract misogynistic commentary or reinforce stereotypical and highly sexualised portrayals of women, which were often widely circulated. Identity‐related posts were frequently described as attracting debate and antagonistic comments, which participants perceived algorithms to further amplify because of the high levels of interaction they generated. Participants commonly perceived younger adolescents to be particularly vulnerable to repeated exposure to misogynistic or hate‐related content. Several reflected that exposure to identity‐related hostility had felt more destabilising at younger ages, before they had developed a more secure sense of self, suggesting that the risks associated with identity‐based feeds may be particularly acute during earlier stages of identity formation.

There's definitely a lot more where the videos are positive things, but the algorithm is also designed in such a way as to entice a little bit of conflict because that gets people engaged… So, if I go into the comments for videos that have a trans theme, there are, more often than not, loads of people just being horrible to each other.

Gender diverse, 17 years

Misclassification via hashtags further complicated this process, with platforms suggesting harmful or oppositional content, alongside affirming content, simply based on shared labels rather than the material's nature and meaning.

*There's not a large queer community at school, I was the only trans kid in a school of a thousand kids. So it was nice to have that online reassurance that there are other people out there, but there was the other problem of when people would make homophobic content they would use the same hashtags. They would use #gay #lgbt and occasionally TikTok doesn't understand the content of the video, it just sees the hashtags… Then I'd interact with the homophobic content like I do and comment, ‘this is stupid, why are you saying this?’ And it would bring up more with the same hashtags.*

Male, 18 years

As a result, identity‐based feeds appeared to be experienced as emotionally mixed spaces, where affirmation, community, hostility, and prejudice co‐existed. Some participants reflected on the mental health risks of algorithmically driven repeated encounters with prejudice.

### 3.4 Mental Health Content as Validation and Spiral

Mental health content was reportedly experienced as highly dependent on timing and emotional state or mood fluctuations. Some participants suggested that algorithmic recommendation sometimes failed to adapt accordingly, continuing to suggest content relating to self‐harm or eating disorders even after they had ‘recovered’. Participants described how content about emotions, coping, or recovery could provide validation, reassurance, and a sense of shared experience during periods of distress.

When you're struggling mentally, it's very easy to feel like you're alone in that situation, I feel like it's very easy to put yourself in a little hole and then just be like you're avoiding the world… Being able to see that other people experience that, through social media, I guess is really helpful… Even if you're stuck in your room, looking on your phone, you're still going to see that other people have that as well.

Female, 18 years

One participant reflected that self‐harm content felt validating at age 13 but they now recognised this as having been harmful – and triggering when it continued to appear years later – illustrating how the same content type may function very differently depending on developmental stage. Several participants described developing mental health literacy as they got older, involving increased awareness of which types of content were helpful, neutral, or potentially triggering for them personally. This was highlighted particularly with reference to self‐harm or restrictive eating content.

[When I was younger] I thought that [self‐harm, suicide and eating disorder content] was what I had to see, so I would interact with it. So I might like it or follow those people because I thought I wanted to be like them… Whereas now, if I saw that. I would press ‘not interested’ or scroll right past it.

Female, 17 years

Rather than being acquired through formal education, this literacy was typically described as developing through trial and error, often following distressing or overwhelming online experiences, and included recognising early signs that certain content was no longer supportive. In some cases, this awareness enabled active strategies such as disengaging, using platform tools, or deliberately reshaping their feeds. Sometimes, as engagement with positive or helpful mental health content accumulated, the same content could become repetitive, intrusive, or distressing, particularly when users' emotional states changed.

I think the more sad and damaged your headspace was the more you'd interact with those [self‐harm or eating disorder] videos because they felt more relatable. If it's a TikTok about hating yourself, you might like it because you're like, “Yeah, I relate to that,” and then more things will get pushed into your algorithm. Whereas, if you're in a happier headspace, you might scroll past it because you're like, “I don't want to think about that right now.”

Female, 17 years

Participants described how seemingly benign or supportive trends could gradually shift into recommendation of more graphic or distressing material as engagement accumulated, with algorithms treating all interaction as ongoing interest.

This… was just really triggering. I was scrolling on my For You page and then there was this trend that was like, “You drew stars around my scars.” It's a Taylor Swift song and it would just be people sharing their self‐harm scars and their tattoos over it. I…kept interacting with it because it was a really cute trend but then it got to the point where people started posting their fresh self‐harm wounds and I was like, “No.” I was not comfortable with that so… I just clicked ‘not interested button’ and I just didn't want to watch that anymore.

Female, 18 years

This dynamic highlighted how timing, emotional state, and recovery stage were critical to how mental health content was experienced, yet largely invisible to algorithmic systems. As a result, algorithms could continue recommending content that reflected previous patterns of engagement rather than adolescents' current needs or wellbeing. Over time, validation or mental health support could transform into spiralling exposure or feeling overwhelmed. These accounts suggest that online content may become incorporated into adolescents' evolving coping strategies, although not always in helpful ways.

### 3.5 News and Activism Content as Moral Responsibility and Burden

News and activism was a content type marked by tension between civic engagement and emotional saturation. Algorithms were perceived to prioritise distressing or emotionally charged stories, amplifying content related to conflict, injustice, or crisis due to its high engagement potential. Some participants raised concerns that repeated exposure to distressing conflict‐related content or graphic violence could become increasingly routine or emotionally numbing over time. Some participants made explicit developmental distinctions about the appropriateness of distressing content for different age groups, reflecting on their own changing capacity to process such material over time.

Participants described feeling pressure to witness, respond to, or support causes they cared about, even when repeated exposure felt overwhelming.

…obviously it's kind of scary and it's awful but at the same time… it's better to know about it and to have to face the fact that it's awful and horrible than to be ignorant of it… even if it does create negative emotions because I mean that's the reality of growing up and that's the reality of life…but there's a difference like in life between what you should see when you're kind of 13 and what you should see when you're 25… sometimes I get kind of overwhelmed by the scale of what's going on in the world. So it would be less one video like this [that would make me feel overwhelmed] but sometimes if I get quite a lot of them.

Female, 17 years

For some, engagement with these issues appeared connected to developing values, empathy, and civic identity. Several participants positively framed interacting with such content as their moral responsibility. However, they described accompanying feelings of guilt, helplessness, or fatigue. This content type also seemed to shape trust and mistrust in institutions and information sources, as participants described navigating uncertainty about credibility, authenticity, and the motivations behind content circulation.

So this is one of the videos of someone who's kind of raising funds for a family in Palestine… I tend to engage with these videos… And because of that, the algorithm obviously then feeds me more and that's something that I'm happy doing because I think, you know, if that's a way of boosting the campaign and helping them, I will do that, but I think it is kind of sad that I don't trust it.

Female, 17 years

Through algorithmic prioritising of distress, news and activism content became difficult to disengage from. At the same time, participants also emphasised the perceived benefits of staying informed, describing news exposure as supporting awareness, critical thinking, and a sense of connection to the wider world. Keeping up to date was framed not only as a moral obligation but as part of developing social understanding and civic identity, suggesting that distress and value often co‐existed within the same encounters with news content.

### 3.6 Body Image as Persistent Comparison

Body image content operated as a particularly pervasive and persistent type, characterised by reported resistance to critical awareness. Participants described how appearance‐related imagery (including body or beauty ‘ideals’) surfaced repeatedly across diverse content types, including travel, fitness, food, commercial or everyday lifestyle posts, making comparison difficult to avoid even when body image was not the focus.

Whilst I might not necessarily think I'm focusing on her, and what she is wearing and what she looks like, I also know that everybody that I see on social media, and out and about in actual life, they're all forming clusters in your mind, of what you should look like whilst you're on holiday… In the moment, I probably wouldn't think I'm focusing on it, but I do know that secretly it's stored away in my mind of what you should look like when you're on holiday.

Female, 19 years

Algorithms were perceived to amplify narrow beauty ideals because such content reliably generated attention, embedding appearance norms and reinforcing gendered and identity‐specific exposure. Several participants also reflected on how susceptibility to appearance‐related content appeared to change with age and maturity. Younger adolescents were perceived to be more vulnerable to internalising appearance norms promoted through social media, particularly before developing stronger critical awareness of edited or idealised imagery. Some participants reflected on their own early social media use (in one case aged nine), before they had the critical resources to evaluate what they were seeing, and one expressed concern for his younger sister and her friends (aged 15) who were engaging with diet and weight loss content and described as developmentally vulnerable. Even in later adolescence, participants often articulated strong awareness that images were edited, filtered, or unrealistic, yet found it difficult to avoid an emotional impact.

I felt like it was what I was seeing was affecting like my mental health and I was like really struggling a lot. The stuff I was seeing ‐ pretty girls, I'd compare myself to them. Like the women are wearing makeup, they don't actually look like that, they might have filters on it or might be edited. I think to an extent I still do compare myself to them… because they still do look pretty even if I know they've got makeup on or there's a filter on it or something. Like sometimes I know they will have lip filler or a nose job or whatever but it does have a big impact on me.

Female, 17 years

This resistance suggested that simply understanding how social media works was not always enough to prevent appearance ideals from being internalised. Participants often reflected that appearance‐related information was absorbed implicitly during everyday scrolling, even when appearance was not the focus of their attention and they consciously recognised images as edited or unrealistic. These accounts suggest that algorithmically repeated exposure to appearance ideals may influence wellbeing through cumulative, often unintended processes of comparison and norm formation. This may be particularly significant during adolescence, when self‐concept and sensitivity to appearance‐based evaluation are still developing.

### 3.7 AI‐Generated Content as Confusion and Mistrust

AI‐generated content formed a relatively novel, low‐trust content type within participants' feeds. This included images or videos created or edited using artificial intelligence, which participants often described as confusing, meaningless, or unsettling. Several reported difficulties distinguishing AI‐generated content from real imagery when scrolling quickly, raising concerns about misinformation.

It's concerning that sometimes it takes you a minute to realise that that's not real… if you weren't paying attention, it would be quite easy to just go ‘oh my god, that's a fact,’ and then just move past.

Female, 16 years

Participants perceived algorithms as promoting AI‐generated content because it was novel, visually striking, or likely to attract engagement, even when they regarded it as irrelevant, low quality, or lacking authenticity. Some described the imagery as creepy or confusing, particularly when it appeared almost, but not quite, human.

It's like uncanny valley. It's not made by a human, it doesn't quite look human, it doesn't quite look real. It's very strange.

Female, 18 years

Participants also raised ethical concerns about AI content, including the environmental impact related to water and energy usage, and the replacement of human creativity, suggesting awareness that extended beyond immediate personal experience. Several participants described AI content that obscured meaning and intent, such as Italian brainrot, which was seemingly humorous but contained offensive or anti‐Muslim messages embedded in the Italian text. Another participant observed that AI threatens to create body image harms through unrealistic generated bodies that users may not recognise as fake.

You can think it [Italian brainrot] is harmless, but… deeply rooted in the entertainment stuff is really serious themes.

Male, 16 years

Many participants reported actively avoiding interaction with AI‐generated content, yet felt frustrated that it continued to appear in their feeds. Overall, AI‐generated content was experienced as a low‐trust type, characterised by uncertainty about authenticity, discomfort with algorithmic amplification, and concern about broader societal implications.

## 4 Summary

Across content types, participants described how algorithmically structured exposure shaped their experiences in ways that appeared particularly salient to key developmental concerns of adolescence, including appearance, peer norms, identity, emotional coping, and civic engagement. Rather than being experienced as isolated encounters, content was commonly described as accumulating through repeated recommendation, with experiences evolving over time as adolescents' emotional states, developmental stage, and contexts changed. Content that was initially engaging, informative, or supportive could become associated with pressure, comparison, emotional burden, or self‐regulatory challenges through ongoing exposure. Perceived impacts therefore depended on how content was repeatedly encountered, interpreted, and managed within everyday algorithmically curated feeds. These patterns form the basis of the discussion that follows, which considers their implications for adolescent development and wellbeing.

## 5 Discussion

In this qualitative study of 27 adolescents, we found that algorithmically recommended feeds comprised diverse content types that were consistently experienced as double‐edged, offering both benefits and risks. Participants described how repeated exposure to similar content, which they often attributed to algorithmic recommendation, shaped how these experiences accumulated over time. Perceived impacts on wellbeing were influenced by contextual factors including emotional state, developmental stage, and individual differences in social media and mental health literacy. Younger adolescents were perceived to be particularly vulnerable, reflecting earlier stages of development in critical evaluation, emotional regulation, and digital coping strategies, while more developed critical awareness appeared to support more selective and reflective engagement with content.

Many of the experiences described by participants appear closely connected to the developmental processes of adolescence. Participants often reflected retrospectively on earlier stages of development, identifying particular vulnerabilities in early or pre‐adolescence, especially in relation to critical evaluation, maladaptive coping strategies, and perceptions of norms relating to body image, restrictive eating, or self‐harm. Content relating to identity, appearance, peer approval, and emotional coping may be especially salient during this period because adolescents are actively developing a sense of self, becoming more responsive to social evaluation, and strengthening capacities for self‐regulation and critical reasoning (Blakemore 2019; Orben et al. 2024). Participants also drew explicit developmental distinctions about the appropriateness of distressing content at different ages, reflecting on their own changing capacities for critical evaluation and emotional processing. This is consistent with evidence that regulatory and evaluative capacities continue to develop across adolescence, and suggests that vulnerability to algorithmically curated content may be particularly acute during earlier developmental stages before these capacities are established (Blakemore 2019; Orben et al. 2024). Algorithmically curated social media feeds appear to have become a contemporary context in which developmental tasks are negotiated. Our findings suggest that algorithmically curated environments may amplify developmental sensitivities to social evaluation, comparison, and emotional feedback by increasing the frequency and persistence of exposure to identity‐related, and socially and emotionally salient content.

Experiences indicated that recommendation systems shape patterns of exposure to both opportunities and risks across content types. Several cross‐cutting patterns were evident. First, content that felt beneficial in the short term could also carry costs over time. For example, entertaining content often provided relief from boredom, distress, or negative emotions, but was also associated with extended scrolling and displacement of homework, hobbies, or other valued offline activities. Second, participants described forms of normalisation across consumerism, body image, and identity‐related content. Repeated exposure to consumption, narrow appearance ideals, prejudice, or violence could make such material feel routine, while blurred boundaries between advertising and entertainment further normalised consumerism. Body image content was experienced as particularly difficult to resist, even when adolescents recognised images as filtered or unrealistic, consistent with evidence that critical awareness does not necessarily protect against appearance‐based comparison (Hermans et al. 2022). Recommendation systems were also perceived to prolong exposure to mental health or appearance‐related content after a young person's emotional state had changed, sustaining unwanted cycles of exposure. Similar dynamics have been identified in recent work on eating disorder‐related content (Parnell et al. 2025). Third, feelings of moral and emotional burden cut across news, activism, identity, and mental health content. Participants reported wanting to support others or stay informed, but also feeling overwhelmed by repeated exposure to distressing material. Because actions such as liking, sharing, or commenting could increase similar recommendations, attempts to help others sometimes intensified emotional strain. Awareness of these dynamics did not always translate into a sense of control, echoing prior research on algorithmic curation (Swart 2021). Differences in awareness and understanding may shape how adolescents interpret and manage curated feeds, potentially contributing to inequalities in digital wellbeing (Gran et al. 2021). Finally, AI‐generated content cut across multiple categories and was commonly associated with mistrust, confusion, unease, and a desire for more meaningful online experiences.

Our findings of the broadly negative but nuanced impacts of exposure to negative news, body image or self‐harm related content align with prior harm‐focused research (Costello et al. 2023), as well as qualitative studies showing that algorithmic curation of mental health content can be simultaneously validating and overwhelming for young people (Susi et al. 2023; Milton et al. 2023; Kanková et al. 2025). We extend this literature by identifying nuanced risks associated with content types that are not explicitly negative or mental health related (entertainment, identity, consumerism, AI‐generated), whereby algorithmic recurrence may contribute to time displacement as well as normative pressures and moral and emotional burden. Where prior work has often examined single content categories in isolation, our findings indicate that adolescents encounter multiple co‐existing content types that they interpret and respond to in different ways depending on context. This may help explain why adolescents frequently report both positive and negative social media experiences within the same period of use (Sequeira et al. 2025), as algorithmically curated feeds expose them to diverse content types that can simultaneously support, challenge, entertain, and distress.

These findings also align with differential susceptibility perspectives, suggesting that wellbeing effects depend on the interaction between content type, emotional state, developmental stage and exposure patterns rather than content alone (Orben et al. 2024; Valkenburg and Peter 2013; Piotrowski and Valkenburg 2015). This is consistent with systematic review evidence showing that adolescents with pre‐existing mental health conditions may be particularly sensitive to content spirals or poor self‐regulation, with impacts shaped by emotional state, content exposure, and patterns of engagement rather than overall screen time (Kostyrka‐Allchorne et al. 2023). Content types were therefore experienced as neither inherently harmful nor beneficial; impacts depended on emotional state, developmental stage, and the intensity and recurrence of exposure. Our findings also extend recent ecological momentary assessment research (Sequeira et al. 2025; Hamilton et al. 2024; Boyd et al. 2024) by suggesting that adolescents' daily positive and negative social media experiences are shaped by the specific forms of algorithmically recommended content they encounter, and by how repeated exposure interacts with developmental stage, emotional state, and individual interpretation. Understanding the characteristics and recurrence of recommended content may help explain why ostensibly similar social media experiences are interpreted differently across individuals and contexts. Consistent with qualitative work on algorithmic curation more broadly (Perez Vallejos et al. 2021), adolescents' accounts also reflected concerns about privacy, safety, and mistrust arising from opaque recommendation systems.

Our study contributes to a growing literature emphasising that social media screen time should not be the primary measure of interest (Orben 2020). We suggest that further research should examine how different content types are interpreted, navigated, and managed by adolescents in everyday digital environments. We recommend studies use sample sizes large enough to examine sub‐group differences by gender, ethnicity, LGBTQ+ identity, or previous mental health experience, as our findings indicate potentially uneven vulnerability to algorithmic harm, consistent with prior research (Costello et al. 2023). Younger adolescents may be particularly susceptible to risks within algorithmically curated digital environments, because emotional regulation, critical evaluation, and digital coping strategies are still developing (Orben et al. 2024).

Our findings suggest that support for adolescent wellbeing should move beyond restricting screen time alone. Interventions may be more effective when they help adolescents build age‐appropriate skills in emotional regulation, critical evaluation, and active management of content exposure, while recognising that developmental needs and vulnerabilities are likely to differ across adolescence.

Although the adolescents interviewed for this study demonstrated reasonably high levels of algorithmic literacy, this was generally reported to have developed through self‐directed learning or in response to negative experiences (Winstone et al. 2026). Algorithmic literacy is rarely and unevenly addressed in school curricula despite its critical importance in managing exposure to harmful content, particularly given evidence that awareness of algorithmic systems varies substantially between users (Gran et al. 2021). Current policy interest in digital literacy provides an opportunity to embed more sophisticated forms of algorithmic literacy within school curricula. Schools must ensure adolescents are aware of the workings and risks of algorithms in their online experiences, and the tools available to them to manage algorithmic recommendations in a way that protects their mental health and wellbeing (Goodyear et al. 2025; Skeggs and Orben 2025; Winstone 2024). Further research is needed to develop and evaluate school‐based algorithmic literacy interventions, in addition to research on algorithmic literacy strength, patterns and impacts (Costello et al. 2023; Oeldorf‐Hirsch and Neubaum 2025). Platform design changes may also help, including clearer advertising disclosure, greater friction around repeated distressing content, safer defaults for younger users, reduced incentives for conflict amplification, and more visible tools for managing recommendations, consistent with emerging child‐centred design codes (Costello et al. 2023).

### 5.1 Strengths and Limitations

Our study benefitted from strong youth advisory input throughout, ensuring that the study design and interpretation of data were grounded in the lived experience of diverse adolescents. Ecological validity was enhanced by using a photo‐elicitation approach, whereby screenshots were used to stimulate discussion of content types. While findings are limited to a UK sample of self‐selecting adolescents, and formal comparison of participant experiences across ethnicity, sexual orientation or mental health experience was not possible, participants described a broad range of experiences and perspectives. Some experiences of content exposure may not have been captured, including due to possible social desirability effects whereby adolescents may have felt uncomfortable sharing screenshots or discussing socially undesirable content (e.g., misogyny).

The sample was predominantly female, which may influence the types of content discussed (e.g., body image, identity content) and limits conclusions about gendered differences in experiences. Some content types (e.g., body image) may be particularly salient for female adolescents, while others (e.g., fitness or masculinised identity content) may be underrepresented in this sample. Future research should purposively recruit more gender‐balanced samples to examine how algorithmically curated content may be experienced differently across gendered and intersectional identities. Despite this, qualitative work in relatively underexplored areas such as algorithmically curated content exposure can provide theoretically informed insight into adolescents' lived experiences and help identify developmental processes that can be examined in future research using larger and more demographically balanced samples.

Although several participants reflected retrospectively on their experiences as younger users, the wide age range (14–19 years) was not systematically analysed by developmental stage, and future research should examine how experiences of algorithmically curated content may differ across early, middle, and late adolescence.

As a qualitative study, findings reflect participants' interpretations and experiences rather than causal effects, and should be understood as providing insight into how algorithmically curated content is perceived and experienced in context.

## 6 Conclusion

Our findings suggest that adolescents' experiences of social media are shaped by the types of content repeatedly encountered within recommended feeds. Across diverse content types, perceived impacts on wellbeing depended less on any single category of content than on how content was experienced, interpreted, and encountered in everyday life, alongside the developmental and personal contexts in which exposure occurred. Repeated encounters with particular forms of content may be especially salient during adolescence, when identity development, sensitivity to social evaluation, and emotional regulation are still evolving. These findings move beyond debates about whether social media is inherently good or bad for mental health and highlight the need for research, education, and policy responses that address the quality, recurrence, and emotional consequences of content exposure.

## Author Contributions

**Lizzy Winstone:** conceptualization, funding acquisition, writing – original draft preparation, resources, project administration, methodology, formal analysis, investigation, writing – review and editing, data curation. **James Parsonage:** funding acquisition, methodology, formal analysis, investigation, writing – review and editing. **Cassey Muir:** funding acquisition, writing – review and editing, methodology. **Amie Randhawa:** funding acquisition, writing – review and editing, methodology. **Lauren Cross:** funding acquisition, writing – review and editing, methodology. **Georgie MacArthur:** funding acquisition, writing – review and editing, methodology. **Evangelina Asiedu‐Addo:** writing – review and editing, methodology, resources. **Emma Garavini:** resources, writing – review and editing, methodology.

## Ethics Statement

Ethical approval was granted by the University of Bristol Faculty of Health Sciences Research Ethics Committee (ref: 22479). All participants gave informed consent prior to participation, with parental or guardian consent and participant assent obtained for those under 16 years.

## Conflicts of Interest

The authors declare no conflicts of interest.

## Supporting information


Supporting File 1

Supporting File 2

## Data Availability

The data from this study will be available under Restricted access conditions from the University of Bristol data.bris repository.
