# Supplementary material for: Adolescents' Experiences of Social Media Content Types and Wellbeing: A Photo‐Elicitation Interview Study
Source: J Adolesc. 2026 Jul 14;98(7):2503–14. doi: 10.1002/jad.70231 (PMC13643666; doi:10.1002/jad.70231)
Supplement: Supplementary file 1 [file JAD-98-2503-s001.docx]

**Supplementary Table 1. Summary of themes, descriptive codes and illustrative data extracts**

| **Theme** | **Codes** | **Data Extracts** |
| --- | --- | --- |
| **Entertainment as relief and time displacement** | Funny animal content;  Relaxing; Living vicariously; Positive reinforcement; Supports hobbies or interests; Distraction from problems; Excessive screentime; Waste of time; Lose track of time; Brain rot | *We've got this one here, which is a dog who hasn't entirely figured out how the treadmill works, which I just found hilarious. I also get loads of animal ones, as well. Certainly triggers quite a lot of dopamine release in my brain, so that's a positive. Female, 17 years.*  *Out of everything, I'd say probably the bulldog in the helicopter [stands out] because it just looks really cute… Obviously, there are more dogs and things. I always like watching stuff like that because it makes you feel better to see stuff like that. Male, 19 years.*  *[Discussing fame on TikTok] I guess maybe a bit, like, worried for the next generation. I don’t know, I feel like I turned out okay after having access to the internet and that sort of thing, but I didn’t have TikTok…I was mostly on, like, YouTube when I was in primary school, at 10. And I was watching just content for people my age. So, kids having unrestrained access to TikTok, it’s a bit scary, because you don’t know what they’re seeing, like what corners of the internet they might be stumbling upon. Female, 18 years.* |
| **Consumerism as convenience and normalised pressure** | Adverts; Influencer culture; Exaggeration; Commercial pressure; Attention online via engagement; Business models | *I’ve been looking at digital cameras and stuff, because I wanted to get one for my birthday. And because I’ve been looking it up, I start getting loads of, like, camera recommendations on my ‘for you’ page. And, like, I didn’t use to get them before I started looking at, like, getting a camera. Female, 17 years.*  *…when they're just trying to sell me something I don't really want like they're just trying to sell me a pen. I go; I'm not interested. I don't really want a pen. Or maybe you get the occasional random video of like carpets like I’m not really interested in carpets. I'll just put not interested. Male, 15 years.* |
| **Identity content as belonging and conflict** | LGBTQ+ community; Autism; Representation of minoritised groups; Community in comments; Negativity; Discrimination; Misogyny; Hashtag misclassification | *At first, it would just be like you know different ethnicity girls people who necessarily like don't look like me, maybe like white women, celebrities, influencers. But now I've tried to like just look at content of people who look like me so I can relate to that. Female, 17 years.*  *Sometimes, the comments aren’t really positive. Sometimes, they’re like, “Yeah, I feel the same, and I’m not autistic.” Sometimes, it, kind of, makes you feel invalidated, because other people think that they’re the same struggles, so intensity of the struggle. And it makes me doubt myself, like, “Am I actually feeling it? Is it really that bad? Am I just being dramatic? Female, 19 years.*  *I think sometimes people fail to recognise the contributions from different cultures, like Indian culture. I think especially for young people of colour, I know that they can feel quite isolated because of that… that does impact my mental health because it does feel like a lot of the time, my culture is being hidden or just made as someone else’s culture, when in reality it was made by my culture. I think now I have grown into not doing this, but when I was younger, I did feel quite embarrassed by my culture and I did feel like, “Oh, people don’t acknowledge our culture. There must be something wrong with it”… Female, 14 years.* |
| **Mental health content as validation and spiral** | Validating content; Triggering content; Recovery content can be gateway to harm; Misuse of MH diagnoses; Scroll more when depressed; Doom-scrolling; Algorithm-driven spirals; Normalisation of harm | *In a way like it makes me feel a little bit seen. Just because I'm getting content which makes me feel that I'm not alone in the fact that I'm not doing great. Female, 18 years.*  *I think, when it’s genuine [recovery content], it is good. There are two cases where it’s bad. I think when they’re just doing it in a performative way so, they’re not genuinely trying to recover, they just want to get, “Oh, well done, so proud of you.” Also sometimes, even if it’s recovery content, it can still remind you of being in need of help which then, I think, is sometimes worse. Female, 17 years.*  *If as a young person you're getting fed content about self-harm or you're seeing people with really like negative eating habits… Or even if you're kind of a 13 year old and you're seeing content that's geared for kind of 16-year-olds, 17-year-olds, 18 year olds, you know, people just talking about stuff that you probably shouldn't be seeing when you're younger. Female, 17 years.* |
| **News and activism content as moral responsibility and burden** | Keeping informed; Watching as activism or support; Desensitisation to harmful content; Sad reflection of world; Violence; Overwhelming; Political echo chambers; Political topics can be guilt-inducing; Political topics can be distressing | *It's generally my information source for, like, the wider world around me, because there are obviously various news channels on TV, and websites that do show the news, but I find that, more often, people can describe it in certain ways which are better than what TV, or radios, or websites have to offer. Female, 17 years.*  *Really bad, to be honest. I try to interact with as many videos as I can. I can't lie, like seeing that type of content, I get it a lot on TikTok, like a lot. It feels like I can't really do anything and so that makes me feel a bit powerless… I'm basically just like sitting back watching… a house burned down and there's nothing I can do about it. Female, 19 years.*  *Well it [how I feel] depends on what I see. If I see something bad then I feel worse. Like if I see some bad news about war and stuff I feel worse. Male, 14 years.* |
| **Body image as persistent comparison** | Unrealistic beauty standards; Body positivity  Beauty drives engagement; Resistant to SML/MHL; Objectification; Comparison; Self-esteem affects resilience | *I think when people aren’t transparent about the fact they’re using filters or fillers or extreme make-up, and push the narrative that that’s natural and that’s an expectation, then it makes people feel a lot worse about themselves and think, “Why do I not look like that?” Female, 17 years.*  *There's so much on social media about beauty standards. I'd say it's more for girls, but again, with guys, there's a lot of stuff about building muscle and stuff, and being a gym bro and things like that. I ignore it, but I feel like, for a younger person, especially in a poor mental state, it could be very influential and very negative. Male, 19 years.*  *I think definitely [I was affected more] when I was younger. I don’t see it as much now, but yeah, definitely when I was growing up. It would be like trying this new beauty hack to fix this aspect of your body, and I was like, “I didn’t even know that was a problem.” But, yeah, I think I definitely saw a lot more of that when I was younger. I definitely worried about it a lot more, but now I don’t really interact with it or see it very much. Female, 18 years.* |
| **AI-generated content as confusion and mistrust** | Disturbing AI content;  Weird or unnerving content; Misinformation; Mistrust; Blood disgusting or triggering | *There's more AI. They're definitely trying to bring that into more stuff, like you can end up watching things and it's like I know that this isn't real. You know that's not real and it's a bit concerning that sometimes it takes you a minute to realize that that's not real. Female, 16 years.*  *It just looks a bit odd and a bit… I don't know. I can't really make sense of what the picture is. I don't know if you can, but yeah, I don't know, but it doesn't look especially harmful or dodgy. It just looks like something a bit unsettling. Male, 19 years.*  *I've definitely fallen for misinformation before, especially when I was younger I think It's dangerous to not be more open about if you're an opinionated creator or a fact-based creator. Male, 18 years.* |

**Additional data extracts relating to experienced or perceived developmental vulnerabilities across multiple themes**

- *I think it would just be good to talk to [younger children] about how not to believe everything they see online, especially at that age. Because I feel like when you’re at primary school, I think you believe things a lot easier, so yeah. I think it would be helpful. Female, 17 years.*
- *I would say [TikTok and Instagram should] regulate the content better. Because some stuff shouldn’t be shown to people of younger ages yet, before they have the critical thinking skills to scroll away or click the ‘not interested’ button. Female, 17 years.*
- *Potentially [more harmful] for younger people because their minds haven't necessarily developed enough to be able to understand that, “Okay, someone is on social media while, they’re with me, that doesn't mean they're on it all the time.” Female, 17 years.*
- *I think [relationship/body image content] definitely would’ve affected me more when I was younger because, obviously, I wouldn’t have had any sexual experience so I would’ve been really worried about not looking good enough for that, if that makes sense? Female, 17 years.*
- *I felt quite sad [workout plan for a 10-year-old] - especially if it’s saying that they did that when they were 10 - because it almost has a sense of relatability because I also did struggle, at a younger age, with my eating and stuff. I know that, if I were to see that when I was younger, I would’ve seen that as a competition and I would’ve wanted to, almost, be more severe than they were so I might be like, “Oh, but I was struggling when I was nine,” for example. It just creates, like, a competition. Female, 17 years.*
- *I've got a younger sister who is more active on social media than I am, and my mum uses social media quite a lot. I think we talk about it, what we see on social media… I don't know if she'd necessarily say to me, “Oh, I saw this video of some woman who was only eating one meal a day and she looked amazing, so that's what I'm going to do.” Like, we talk about it, but I don't think we necessarily talk about the bad side. Female, 19 years.*
- *Obviously, seeing 40kg, that's unhealthy. So, I think that's damaging, in a way, but obviously I think I understand that, I guess, weight looks different on everyone, but I think it's harmful in younger people, yeah. Female, 19 years.*
- *I wasn’t good at practicing posting safely online either [when I was younger], because I remember posting, like, in my school uniform, and running around, and I had, like, a public account on TikTok, so that was really unsafe. I’ve taken down all the videos, but still, you know, in the back of my head I think about- like, I wouldn’t even post my lanyard for college on there, because it’s just like, I don’t want people to know where I’m going to school, and that was when I was a child, so yeah. Female, 17 years.*
- *I don't get recommended anything that's untoward, but I would say a lot of people with, like, Andrew Tate and stuff, lads younger than me will have massively been influenced by that, wrongly. Male, 19 years.*
